# Preoperative factors influencing success in pterygium surgery

**DOI:** 10.1186/1471-2415-12-38

**Published:** 2012-08-08

**Authors:** Ana Torres-Gimeno, Lucía Martínez-Costa, Guillermo Ayala

**Affiliations:** 1 Department of Ophthalmology, Hospital Dr. Peset, Avda. Gaspar Aguilar, Valencia, 90, E-46017, Spain; 2 Department of Statistics and Operational Research, Universidad de Valencia, Avda Vicent Andrés Estellés, Burjassot, 1, 46100-, Spain

**Keywords:** Risk factors, Sunlight exposure, Pterygium surgery, Conjunctival autograft

## Abstract

**Background:**

To identify preoperative, perioperative and postoperative risk factors that influence the success of pterygium surgery.

**Methods:**

This is a prospective study of thirty-six patients with primary or recurrent pterygia. A detailed anamnesis and an ophthalmological examination were performed looking for the following factors: age, race, latitude and altitude of the main place of residence, hours of exposure to the sun, use of protective measures against UV-radiation, classification of pterygium, width of the pterygium at limbus, surgical technique (conjunctival autograft plus suturing versus tissue glue), graft alterations (misapposition, granuloma, haemorrhage, oedema, retraction or necrosis), and postoperative symptoms (foreign-body sensation, pain). The examinations were performed 2 and 7 days and 2, 6 and 12 months after surgery. In addition, recurrence was defined as any growth of conjunctiva into the cornea.

**Results:**

A logistic regression and a survival analysis have been used to perform data analysis. A total number of 36 patients completed a one year follow-up. A total of 13 patients were born and lived in Spain, and 26 came from other countries, mostly Latin America. A total number of 8 males (no women) presented a recurrence, mainly between 2 and 6 months. The hours of sun exposure through their life was independently related to surgical success. Pterygia of less than 5 mm of base width showed a weak positive correlation with recurrence. None of the other factors considered were significantly related to recurrence.

**Conclusions:**

Male gender and high sun exposure are strongly and independently related to surgical success after the removal of pterygia.

## Background

Pterygium is a wing-shaped, fibrovascular tissue crossing the limbus into the cornea. It is a common ocular surface disease, but also potentially blinding, so different surgical procedures have been used to prevent it. Recurrence after excision remains a great challenge. Nowadays, it is accepted that conjunctival autograft surgery is the procedure of choice for the treatment of both primary and recurrent pterygium [1,2].

The pathogenesis of pterygia is still not completely understood. An overall view of the growth process reveals a multiplicity of factors that are correlated and interrelated [3]. Recent evidence implicates anti-apoptotic mechanisms, immunological mechanisms, cytokines, growth factors, extracellular matrix modulators, genetic factors and viral infections, among other possible causative factors [4,5].

The prevalence rates vary widely (from 2% to 29%) [1], but generally they are higher in the tropics than at temperate latitudes [6,7]. It is accepted that pterygium occurs in an equatorial belt delimited by Latitude 40N and S, associating it with ultra-violet light [7-9]. Prevalence increases geographically towards the equator and is greater in people exposed to outdoor environments [10]. In addition, there are associations with rural regions, increasing age and male gender, which correlate with outdoor work [11]. Although a lot has been written about the risk factors for developing a pterygium, the relationship between them and the outcome of the surgery is still unclear.

The purpose of our study is to identify preoperative, perioperative and postoperative risk factors that influence the success of pterygium surgery.

## Methods

This is a prospective study, involving thirty-six patients with primary or recurrent pterygia, treated at the Dr. Peset Hospital (Valencia, Spain) from September 2007 to July 2008. The ethics committee of the Dr. Peset Hospital CEIC (Comité Ético de Investigación Clínica) has given its approval to the study in compliance with the Helsinki Declaration. The reference number is 8/2005. A total of 13 patients were born and had always lived in Spain. A total of 26 came from other countries, mostly Latin America. A comprehensive medical and ocular history was obtained by a single ophthalmologist (the coauthor AT). The patient ´s age, gender and race (Caucasian or Hispanic) were recorded. Then a detailed questionnaire was undertaken in order to evaluate: principal place of residence (the name of the place where he/she lived most of his/her life), exposure to sun over lifetime (hours per day, during workdays and rest days), use of sun protection (none, hat, sunglasses, both of them) and the use of prescription glasses. We looked for the latitude and altitude of the patient ´s principal place of residence. Furthermore, Snellen visual acuity measurement, applanation tonometry, slit-lamp examination, funduscopy and anterior segment photography were performed pre-operatively. A pterygium was defined as a radially oriented fibrovascular lesion crossing the nasal or temporal limbus. Moreover, the pterygia were graded according to the system used by [12]: grade 1 (atrophic: episcleral vessels under the body of the pterygium are not obscured and clearly distinguished), grade 3 (fleshy: episcleral vessels totally obscured) and grade 2 (intermediate: all other pterygia not falling into these 2 grades). We also estimated the width of the pterygia at limbus, dividing them into two groups: wide base (≥ 5mm) and narrow base (<5 mm). The pterygia sizes were measured with a slit lamp by using a slit beam of light. Tear break-up time measurement, evaluation of ocular motility, presence of symblepharon and previous surgery were also indicated. Inclusion criteria: Patients were included if they presented a primary or recurrent pterygia, for which surgery was recommended considering the following critreria. (i) A visual disturbance either through pupillary aperture invasion or by significantly inducing corneal astigmatism (more than 2 diopters measured by corneal topography and not attributable to other causes). (ii) Documented enlargement over time in the direction of the centre of the cornea. (iii) Chronic symptomatic inflammation (significant foreign-body sensation or pain, hyperemia, dellen, corneal epithelial defect). Exclusion criteria: Subjects with other pathology features or infection of the ocular surface that might alter wound healing, mainly connective tissue disease and diabetes were excluded. All patients gave written informed consent to participating in the study, which was approved by the ethical committee of our hospital. Moreover, the surgical procedures complied with the tenets of the Declaration of Helsinki.

### Surgical technique

The surgical technique used is similar to procedures described earlier. [2,13] Patients were randomized into 2 subgroups: Tissue glue group (TG) and Mersilk group (MG). Tissue glue was used to attach the auto graft in 21 patients and 7.0 Mersilk sutures were used in 18 cases.

Tissucol Duo*®* (Baxter AG, Vienna, Austria) is a fibrin solution that simulates the final stage of the coagulation cascade. The kit includes 2 syringes, one containing a solution comprised of factor XIII, plasminogen, plasma fibronectin and fibrinogen and a second syringe that contains a human thrombin solution. All patients were operated on by the same surgeon (LM). The procedure was carried out under topical and subconjunctival (lidocaine 2%) anaesthetic. Pterygium dissection from the head towards the body was made. Then the pterygium head, along with the underlying tenon tissue, was excised. Episcleral scarring was removed and minimal cauterisation was used to control bleeding in the recipient bed. The area of the conjunctival defect was measured with a caliper, and a free conjunctival-limbal auto graft measuring the same size as the conjunctival defect was obtained from the superotemporal quadrant of the bulbar conjunctiva. For the graft dissection, 2% lidocaine was injected under the conjunctiva in order that only conjunctiva be obtained. The conjunctiva was dissected from the fornix to the limbus, and graft dissection was extended by 0.5 mm into the clear cornea to include the limbal element of the graft. Meticulous dissection was performed in order to remove the Tenon capsule as much as possible. In the suture group, the limbal part of the graft was attached to the adjacent conjunctiva and episclera with 2 interrupted 7-0 Mersilk sutures. In the tissue glue group, one drop of thrombin solution was applied over the bare sclera in the recipient bed and one drop of protein concentrate solution was applied over the stromal side of the graft. The graft was immediately placed in the correct orientation onto the bare. Postoperative therapy included Tobramycin-Dexametasone combination every six hours, Pranoprofen eyedrops every six hours for four weeks and a Povidone artificial tear every six hours for two months. The postoperative follow-up was carried out by a single ophthalmologist (AT). The examinations were performed between 2 and 7 days and between 2, 6 and 12 months after surgery. The anterior segment and the integrity of the autograft (granuloma formation, subconjunctival haemorrhage, edema, necrosis, retraction and gaping or displacement of the graft-bed junction) were evaluated by slit lamp biomicroscopic examination at each visit. Silk sutures were removed at the one week visit. Recurrence was defined as any growth of the conjunctiva into the cornea. All patients were asked about subjective symptoms and graded into 4 groups: asymptomatic, foreign-body sensation, mild pain or severe pain (defined by the need for an oral analgesic). At the 2-month visit, visual acuity was also checked and an anterior segment photograph was taken at the 12-month visit. Reoperation was performed with the patient ´s consent if recurrent pterygium was observed on any follow-up examination, which happened in just two cases. The remaining patients that recurred were free of symptoms and preferred to wait and see evolution.

### Statistical analysis

Statistical analysis has been performed using the statistical software environment R [14]. Logistic regression (the function glm included in [15]) and survival analysis has been used. The interval censored data has been analyzed using [16]. Variable selection has been applied. The method used consists of the minimization of the AIC (Akaike information criterium). It has been performed using the function stepAIC included in the R package MASS [15]. The interval censored data has been analyzed using the R package interval [16].

## Results

Statistical descriptives of the patient ages by taking into account the recurrence are shown in Table 1: the minimum, first quartile (or 25% quantile), sample mean, median, third quartile (or 75% quantile) and the maximum. Those patients that suffered recurrence were slightly fewer than those that did not although this difference was not statistically significant. The p-value in the last column corresponds to the t-test where the mean ages for recurrence and no recurrence are compared. Table 2 shows the odds ratio (with the corresponding confidence interval) between recurrence and the different variables under study (transformed into binary variables). It can be seen that gender is the most important risk factor for surgical outcome. For the remaining variables the confidence interval contains the value one i.e. we cannot reject that there is no association between the variable considered and pterygia recurrence. Let us consider the variable giving us whether the pterygium has recurred after the one year follow-up, the one-year recurrence (1 = recurrence and 0 = non-recurrence). Our major aim will be to study the influence of some covariables over the one year recurrence. The covariables considered are: age (AGE), gender (GENDER: 1, male; 0, female), race (R, Caucasians and Hispanics), latitude (LAT), altitude (ALT), workdays sun exposure (WDE), non-workdays sun exposure (NWDE), primary vs recurrent pterygia (PR), pterygium type (PT: 1, 2 and 3 according to Tan classification), wide base (WB: less or greater than 5 mm), surgical technique (ST: fibrin glue and sutures), miss-apposition (MA: 0, not and 1, yes) and protective measures (PM: 0, none; 1, hat; 2, sunglass).

**Table 1:** Statistical summaries of age where no-rec means no recurrence cases and rec corresponds to recurrence cases meanwhile the p-value corresponds to the t-test where the mean ages for recurrence and no recurrence are compared


**Age**	**Min.**	**Median**	**Mean**	**3rd Qu.**	**Max.**	**N**	**p-value**
All	24.00	45.00	46.21	58.00	77.00	36	
No-Rec	24.00	45.00	47.18	58.00	77.00	28	0.4107
Rec.	31.00	37.50	42.75	49.25	68.00	8	

**Table 2:** Columns “Non recurrence” and “Recurrence” correspond to the number of cases without and with recurrence respectively; the column % Recurrence contains the percentage corresponding to recurrence cases; for each variable (gender, latitude, race, wide base,previous surgery, surgical technique and misapposition), recurrence odds-ratio and corresponding confidence interval [CI] are given


	**Non recurrence**	**Recurrence**	**% Recurrence**	**Odds-Ratio [CI]**
Gender				
Female	23	0	0	72.64
Male	5	8	62	[3.62,1458.15]
		(6 hisp. and 2 cauc.)		
Latitude				
[0,37]	16	5	23	1.25
				[0.25,6.29]
[37,100]	12	3	20	
Race				
Caucasian	10	2	16	0.6
				[0.10,3.55]
Hispanic	18	6	25	
Widebase				
< 5 mm	15	5	25	1.44
				[0.29,7.24]
> 5 mm	13	3	19	
Primary/Recurrent pterygia				
Primary	23	5		2.25
				[0.38,13,16]
Recurrent	5	3		
Technique				
Glue	14	5	26	1.66
				[0.33,8.35]
Suture	14	3	18	
Misapposition				
No	8	2	20	0.83
				[0.14,5.03]
Yes	20	6	23	

A logistic regression has been applied where the binary response is the one-year recurrence. The covariables remaining in the model after variable selection are GENDER, WDE, NWDE and WB. Table 3 shows the parameter estimates and the p-values testing a null coefficient.

**Table 3:** Estimates of the coefficients and p-values observed in the reduced model (see text for details): WED = workdays sun exposure, NWDE = non-workdays sun exposure, WB = wide base of the pterygium


	**Coefficient**	**p-value**
Intercept	0.019	0.844
GENDER	0.590	<0.001
WDE	0.069	<0.001
NWDE	-0.045	0.012
WB	-0.137	0.129

**Figure 1:**
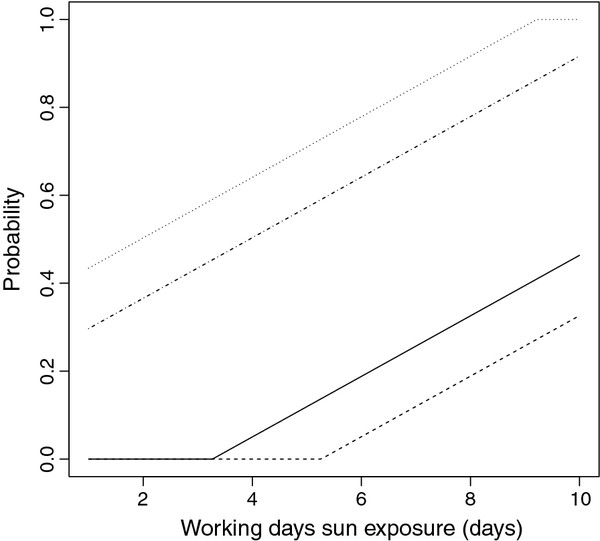
**Probabilities of one year follow-up equal to one for different values of working days sun exposure (WDE).** From top to bottom, we have male with narrow base (dotted line), male with wide base (dotted dashed line), female with narrow base (solid line) and female with wide base (dashed line).

**Figure 2:**
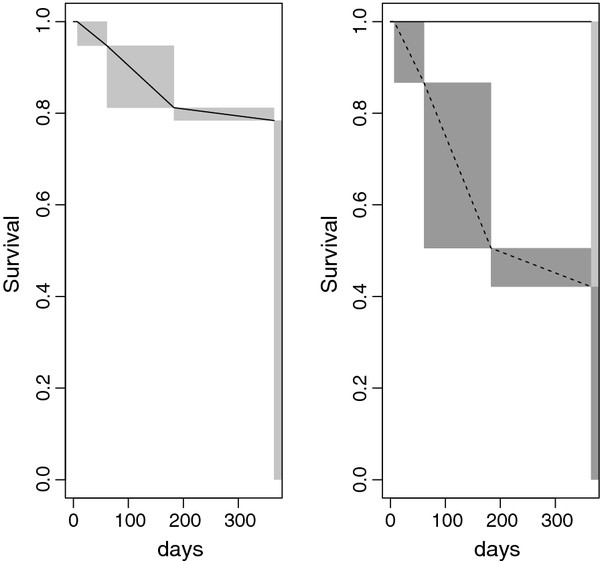
**Left: Survival function estimated i.e. the proportion of non-recurrence cases.** Right: Estimated survival functions for male (dashed line) and female (solid line). The abscissa corresponds to days after surgery.

Figure 1 displays the probability of recurrence during the one year follow-up for different values of WDE (from one to ten hours/day of sun exposure on workdays), NWDE equal to the mean value observed, and the variables GENDER and WB. Figure 1 shows four lines that from top to bottom correspond to male without wide base, male with wide base, female without wide base and female with wide base. Note that the main differences correspond to gender. The differences due to WB are clearly smaller. For each eye our protocol gives us the time from surgery to recurrence. In fact, we are dealing with interval censored data because for each patient the time interval when recurrence appears is known. We have checked each patient at days two and seven, two months, six months and one year. This interval censored data has been used to estimate the survival function S(t) (for each time t, the fraction of patients with a time to recurrence greater than t). Figure 2 (left) displays the survival function estimated. The recurrences mainly appear between 2 and 6 months after surgery. The interval censored survival times have been compared (log-rank two sample test) for gender and WB. A significant p-value is observed for gender (p<0.001) and non significant for WB (p=0.64). Figure 2 (right) displays the estimated survival functions considering the gender. A total of thirty-six patients completed the 12-month follow-up period and eight of them (22%) presented a recurrence within a year post-operatively. The most important variable influencing surgical success is gender. All patients that suffered a recurrence were male. The second most important variable is the hours that the subject was exposed to sun radiation, mainly during workdays, but also on non-workdays. Patients that recurred were younger than those who did not recur (but not statistically significant).

No clear relationship has been found between the recurrence and protective measures (PM), race (R), altitude (ALT) and latitude (LAT) of the principal place of residence, pterygium type (PT), primary-recurrent pterygia (PR), surgical technique (ST) and miss-apposition (MA). Only a narrow base (less than 5 mm) pterygia showed a weak positive trend to recurrence.

## Discussion

Pterigia is more common in men than in women [10,11,17-19]. The female gender has been reported as a marker for lower occupational or recreational exposure to sunlight. However, greater exposure to the sun alone cannot explain the male preponderance to developing pterigia. It is suggested that other unknown factors probably play a role [18,20]. In a paper comparing the outcomes of pterygium surgery, males and patients under 40 years of age face a greater risk of recurrence [21]. Our results indicate that the male gender is also strongly and independently associated with pterigia recurrence after surgery. Surprisingly, a younger age does not mean a greater risk of recurrence in our cases.

Epidemiological factors influencing pterygium develop- ment have been suggested (chronic sun exposure, peri-equatorial place of residence, high altitude or dry weather) [7,17,18,22,23]. Prevalence increases geographically towards the equator and sun exposure has been reported as one of the most important factors influencing pterigyum development [10,17,24-28]. Therefore, keeping the eyes out of direct sunlight has been defended as beneficial. Measures such as wearing sunglasses or prescription glasses, have been described as protective factors against pterygium development [7,24,26,29].

In a retrospective work, 21 hispanic ethnicity has been reported as a potentially important risk factor for the recurrence of primary pterygia treated with conjunctival auto graft. Other important factors such as hours of sun exposure were not considered.

In Spain, the immigration rate has increased considerably in the last years, until 2008, so all of our patients spent most of their lifetime in their respective countries. This circumstance permits us to compare the success of pterygium surgery depending on some epidemiological factors. Clinicians observe that in Spanish people pterygia often develops after the forth decade of life, mainly in outdoor workers, and have an atrophic appearance. However, pterygia in immigrants that came mainly from the peri-equatorial countries of Latin America appear at a younger age and have a more aggressive aspect. We have not found a significant relationship between ethnicity, latitude and altitude of the main place of residence and surgical recurrence. Protective measures against sun radiation, such as wearing sunglasses, refractive glasses or a hat do not affect the recurrence ratio in our sample either. We believe that this is a factor which is difficult to evaluate over an individual ´s life. However, sun exposure has been the second most important factor influencing recurrence. Strong epidemiological evidence links exposure to ultraviolet and visible light with the development of pterygium. It has been proposed that the focal limbal irradiation of basal epithelial cells results in the alteration of these cells and a breakdown of the limbal barrier [30]. Our results indicate that focal limbal insufficiency due to high sun exposure for years also determines a higher probability of recurrence after conjunctival grafting.

In our study, narrow base pterygia (less than 5 mm at the limbus) showed a weak association with recurrence. This factor is not usually considered in pterygia studies. Larger prospective studies should be undertaken to confirm this. Conjunctival autografting is often utilized with low recurrence and good success in both primary and recurrent pterygia. Some surgeons perform grafting as their standard procedure for the treatment of both primary and recurrent pterygium. This is due to a low recurrence rate, efficient limbal reconstruction and long-term safety compared with other techniques such as mitomycine application and beta radiation [1,2,31]. Some authors use a temporal amniotic membrane patch covering the excised area with low recurrence ratio [13]. The auto graft can be fixed with sutures or fibrin glue. The use of a tissue adhesive simplifies the surgical technique and minimizes postoperative inflammation, reducing both operating time and postoperative pain [32]. What is more, it provides an excellent hemostasis even in congested eyes with recurrent pterygium [33]. The recurrence rates when using this surgical technique, vary from 2% to 34%, depending on individual technique and surgical experience [1-12]. In addition, we believe that the follow-up period must be at least one year after surgery in order to detect all recurrences. When comparing sutures to fibrin glue, most of the studies show a low recurrence rate when the graft is attached with glue ([1,2,34-36]), or at least similar rates with both procedures. 37-39 Besides, the glued-graft also reduces surgery time and improves postoperative patient comfort [1,34-39]. We have not found any difference in the recurrence ratio between both procedures. In addition, depending on postoperative alterations in the graft (granuloma, edema, haemorrhage, retraction, misapposition or necrosis), they showed no relationship with recurrence ratio either.

## Conclusion

Epidemiological and clinical aspects influencing pterygia development have been extensively studied, but few works consider those factors related to surgery failure. Our results indicate that male gender and sun exposure are strongly and independently related to surgical success after pterygia removal. We believe that sun exposure over life and male gender should be considered as additional risk factors for recurrence after pterygium autograft. Extremely meticulous surgery and close follow up must be employed when operating these patients. We are aware about the small sample sizes. We have a small number of patients evaluated. Clearly, this is a limitation of our paper and consequently we have a small statistical power. Larger studies should be carrier out in order to confirm our results.

## Competing interests

The authors declare that they have no competing interests.

## Author’s contributions

ATG and LMC conceived the study and collected the data. GA perfomed the statistical analysis. All authors read and approved the final manuscript.

## Pre-publication history

The pre-publication history for this paper can be accessed here:

http://www.biomedcentral.com/1471-2415/12/38/prepub
